# Supplementary material for: Resolving the full spectrum of human genome variation using Linked-Reads
Source: Genome Res. 2019 Apr;29(4):635–45. doi: 10.1101/gr.234443.118 (PMC6442396; doi:10.1101/gr.234443.118)
Supplement: Supplemental Material [file supp_gr.234443.118_Supplemental_longranger-2.2.zip › longranger-2.2.tar.bz2!/lr2.2-public/lib/stan/cmdstan/stan/doc/api/html/check__greater_8hpp_source.html]

Stan: src/stan/math/error\_handling/check\_greater.hpp Source File


|  |  |
| --- | --- |
|  | Stan  2.5.0  probability, sampling & optimization |


- Main Page
- Related Pages
- Namespaces
- Classes
- Files

- File List
- File Members

All Classes Namespaces Files Functions Variables Typedefs Enumerations Enumerator Friends Macros Pages

- src
- stan
- math
- error\_handling

check\_greater.hpp

Go to the documentation of this file.

1 #ifndef STAN\_\_MATH\_\_ERROR\_HANDLING\_CHECK\_GREATER\_HPP

2 #define STAN\_\_MATH\_\_ERROR\_HANDLING\_CHECK\_GREATER\_HPP

3

4 #include <stan/math/error\_handling/dom\_err.hpp>

5 #include <stan/math/error\_handling/dom\_err\_vec.hpp>

6

7 namespace stan {

8  namespace math {

9

10  namespace {

11  template <typename T\_y,

12  typename T\_low,

13  typename T\_result,

14  bool is\_vec>

15  struct greater {

16  static bool check(const char\* function,

17  const T\_y& y,

18  const T\_low& low,

19  const char\* name,

20  T\_result\* result) {

21  using stan::length;

22  VectorView<const T\_low> low\_vec(low);

23  for (size\_t n = 0; n < length(low); n++) {

24  if (!(y > low\_vec[n]))

25  return dom\_err(function,y,name,

26  " is %1%, but must be greater than ",

27  low\_vec[n],result);

28  }

29  return true;

30  }

31  };

32

33  template <typename T\_y,

34  typename T\_low,

35  typename T\_result>

36  struct greater<T\_y, T\_low, T\_result, true> {

37  static bool check(const char\* function,

38  const T\_y& y,

39  const T\_low& low,

40  const char\* name,

41  T\_result\* result) {

42  using stan::length;

43  VectorView<const T\_low> low\_vec(low);

44  for (size\_t n = 0; n < length(y); n++) {

45  if (!(stan::get(y,n) > low\_vec[n])) {

46  return dom\_err\_vec(n,function,y,name,

47  " is %1%, but must be greater than ",

48  low\_vec[n],result);

49  }

50  }

51  return true;

52  }

53  };

54  }

55

56  // throws if any element in y or low is nan

57  template <typename T\_y, typename T\_low, typename T\_result>

58  inline bool check\_greater(const char\* function,

59  const T\_y& y,

60  const T\_low& low,

61  const char\* name,

62  T\_result\* result) {

63  return greater<T\_y,T\_low,T\_result,is\_vector\_like<T\_y>::value>

64  ::check(function,y,low,name,result);

65  }

66

67  }

68 }

69 #endif

dom\_err.hpp

stan::math::dom\_err

bool dom\_err(const char \*function, const T &y, const char \*name, const char \*error\_msg, const T\_msg error\_msg2, T\_result \*result)

**Definition:** dom\_err.hpp:36

stan::math::check\_greater

bool check\_greater(const char \*function, const T\_y &y, const T\_low &low, const char \*name, T\_result \*result)

**Definition:** check\_greater.hpp:58

stan::length

size\_t length(const T &)

**Definition:** traits.hpp:159

stan::math::dom\_err\_vec

bool dom\_err\_vec(const size\_t i, const char \*function, const T &y, const char \*name, const char \*error\_msg, const T\_msg error\_msg2, T\_result \*result)

**Definition:** dom\_err\_vec.hpp:33

dom\_err\_vec.hpp

stan::get

T get(const T &x, size\_t n)

**Definition:** traits.hpp:143


---

[ Stan Home Page ]

*© 2011–2014,
Stan Development Team.*
